# Metalloproteome plasticity — a factor in bacterial pathogen adaptive responses?

**DOI:** 10.1042/ETLS20230116

**Published:** 2024-02-07

**Authors:** Alastair G. McEwan

**Affiliations:** Australian Infectious Diseases Research Centre, School of Chemistry and Biosciences, The University of Queensland, St Lucia 4072, Australia

**Keywords:** bacteria, host–pathogen interactions, metalloenzymes

## Abstract

Through homeostatic processes, bacterial cells maintain intracytoplasmic metal ions at concentrations which enable the ‘correct’ metal to be inserted into an enzyme, thereby ensuring function. However, fluctuations in intracytoplasmic metal ion concentrations mean that under different conditions certain enzymes may contain different metals at their active site. This perspective describes examples of such cases and suggests that metalloproteome plasticity may contribute to the dynamic adaptation of pathogens to stresses in the host environment.

## Introduction

Metal metabolism plays a critical role in host–pathogen interactions. Bacterial pathogens have evolved a variety of processes to acquire transition metals, particularly Fe, Zn and Mn from the host while nutritional immunity is now recognized as a key component of the host innate immune response at the cellular and systemic level that can withhold metals from bacterial pathogens [1]. It is also established that metals in excess can be toxic, and so the acquisition of metals is balanced through homeostatic processes to ensure that overload does not occur [2]. It is usually the case that enzymes function with only one type of metal under normal physiological conditions but in some cases, it appears that there can be redundancy of metal usage [3]. This *Perspective* discusses the dynamics of metal availability and ways in which bacterial adaptive responses may contribute to pathogen survival in the host through metal interchangeability within enzymes and proteins.

## Protein metalation and intracellular metal availability in bacteria

The Irving–Williams series of metal–ligand stability [4] provides the framework for understanding intracellular metal availability. For those metal ions relevant to this perspective affinity of metal binding to a biomolecule follows an order from Mg^2+^, the weakest binding ion, through the first row transition metals in the order Mg^2+ ^< Mn^2+^ < Fe^2+^ < Co^2+^ < Ni^2+^ < Cu^2+^ (and Cu^+^) > Zn^2+^, with Cu^2+^ (and Cu^+^) being the tightest binding ion in the series. This means that under conditions where available concentrations of these metal ions were the same proteins would be loaded with the metal with the highest affinity (i.e. Cu^2+^ and Cu^+^) and occupancy by weaker binding metals would only occur once the supply of the highest affinity metals was exhausted. To manage this chemical actuality biological systems have evolved homeostatic processes that ensure that cytoplasmic ‘free' metal ions are held at concentrations that are the inverse of the Irving–Williams series [5,6]. Thus, ‘free' Cu^+^ is typically held at around a concentration of 10^−15 ^M or less while Mg^2+^ is much higher at ∼10^−3 ^M [7], with the concentration of other transition metal ions lying between these values. These metal ion homeostatic systems enable the ‘correct’ metal ion to be inserted into an enzyme or protein, thereby enabling biological function. Through systematic studies, Robinson and co-workers [5] established that the set points for cytoplasmic ‘free' metal ions (also referred to as metal availabilities) are determined by metal buffering molecules and transcriptional sensors which are tuned to detect the activity of these metal complexes and alter the expression of metal ion transporters (efflux or import) and metal ion binding and storage proteins.

## Interchangeability of manganese and iron in metalloproteins

Manganese is found in ∼8% of metalloproteins as indicated by the MACiE database [8] and this compares with 40% for magnesium. Although enzymes containing iron represent about a quarter of metalloenzymes, only a small proportion of these are mononuclear. In an analysis of six EC classes of enzymes containing metals Mg was found to be distributed across all classes and was predominant in lyases and transferases while Zn was predominant in hydrolases where its Lewis acid properties are effective [8]. However, it is quite often the case that purified enzymes exhibit activity with more than one metal ion, and the interchangeability of Mg and Mn has been described [9]. In this context, Fe is rarely considered since the instability of ferrous iron in the air would mean that it would not be the divalent ion of choice for biochemists for reconstitution of enzyme activity and so its presence in mononuclear metalloenzymes may be underestimated. For the majority of enzymes, the MACiE data base provides a clear categorization of the metalation state but for some enzymes, the situation *in vivo* may be more complex.

Intracellular available Mn^2+^ and Fe^2+^ are both held at ∼10^−6^ M, although the total intracellular concentration of Mn^2+^ is higher than Fe^2+^, as predicted by theory [5]. Iron is very often in high demand by bacterial cells and specific use of this metal occurs through the formation of iron–sulfur clusters and haem cofactors that are central to respiratory enzyme function [10]. Mn^2+^ cannot substitute functionally for Fe^2+^ in these prosthetic groups, although it is interesting to note that some of the toxic effects of Mn^2+^ overload relate to the inhibition of ferrochelatase [11] and thus haem biosynthesis. However, there is also the potential for functional interchangeability of Mn^2+^ and Fe^2+^ in some enzymes. In many organisms, Mn-specific (SodA) and Fe-specific (SodB) superoxide dismutases only function with their cognate metal ion but there are ‘cambialistic’ enzymes (SodM), where either Fe or Mn can be present in a functional enzyme [12]. Another example of interchangeability is in some mononuclear metalloenzymes, exemplified by ribulose-5-phosphate epimerase (Rpe) [13]. It was shown that in *Escherichia coli*, Mn^2+^ uptake in response to peroxide stress provided protection through the formation of a Mn^2+^-metalated Rpe rather than a Fe^2+^-metalated enzyme. This led to a proposal that many mononuclear metalloenzymes may operate as Fe^2+^-enzymes under anaerobic conditions but under conditions of oxidative stress (or more generally under aerobic conditions), a Mn^2+^-enzyme is formed that is protected against oxidative damage. The number of enzymes in this category is still unclear, although it has been suggested that peptide deformylase, cytosine deaminase and threonine dehydrogenase may be in this class [13].

The interchangeability of Mn^2+^ and Fe^2+^ in *E. coli* is conditional since expression of the Mn^2+^ transporter, MntH, is controlled by OxyR, which means that significant Mn^2+^ import in this bacterium, only occurs in response to peroxide stress. In *Salmonella enterica* sp. Typhimurium nitrosative stress appears to be a key driver of Mn^2+^ uptake through increased expression of the MntH symporter and the SitABC primary transporter [14]. This change in expression is mediated via the MntR de-repression and Fur de-repression of *sitABC* and *mntH* expression, that is consistent with a reduction in intracellular ‘free’ iron and manganese limitation. In this adaptive response in *S.* Typhimurium elevation of Mn^2+^ makes use of the antioxidant properties of this ion, while reduction in the use of Fe^2+^ abrogates the pro-oxidant effects of this metal.

## Interchangeability of manganese and magnesium in metalloproteins

As observed by Kehres and Maguire [15] enzymes where Mg and Mn is interchangeable seem to be clustered in intermediary carbon metabolism, particularly in enzymes in what is known as the PEP–pyruvate–oxaloacetate (PPO) node which acts as the metabolic link between glycolysis/gluconeogenesis and the TCA cycle [16]. Enzymes containing Mn may have a higher catalytic efficiency compared with the same enzyme with Mg [9] but it remains an open question as to whether this has a significant physiological impact. One possibility is that the efficient metabolism of pyruvate is important during growth on non-fermentable substrates such as short-chain fatty acids or l-lactate. Recently, it has been shown that l-lactate production in M2 macrophages promotes the growth of *S.* Typhimurium [17]. Potentially, this could be associated with changes in Mg/Mn ratio since it is known that the Slc11a1 (NRAMP 1) transporter in macrophages may export Mg from the phagosome, thereby limiting this ion. Mg limitation triggers a stress response in *S.* Typhimurium through the action of the PhoP/Q system but another consequence might be to decrease the Mg/Mn ratio in the intracellular pathogen [18,19]. This may be enhanced by the triggering of Mn uptake by *S.* Typhimurium under conditions of nitrosative stress, as described earlier [15]. Metal limitation also appears to be imposed on *S.* Typhimurium growing in epithelial cells as indicated by the elevated expression of proteins involved in Mn, Fe and Zn acquisition, and Mg limitation is also inferred through the increased expression of PhoPQ [20]. There are a range of additional protective effects that Mn could confer on the stressed bacterial cell and one of these may be maintenance of ribosome integrity through substitution for Mg and protection from oxidative damage that can occur from Fe^2+^-mediated rRNA degradation [21].

The situation for *S.* Typhimurium in macrophages indicates a role for Mn in a transient dynamic response to stress imposed by the host but it is the case that some bacteria that exhibit a high dependence on Mn which can accumulate to high intracellular concentrations above the usual set point. Achieving this would require an intracellular sink for Mn and it is suggested that the ribosome is potentially a site where Mn is accumulated in a form that might not be rapidly exchangeable. Given the central role of ribosomes in relation to cell growth [22] this may provide a link between Mn and broader physiological responses that this ion is known to influence [23].

## Conclusions

Although many dimensions of the functional role of transition metal ions in host–pathogen interactions are now well established there is still much to uncover regarding the way in which dynamic changes in these metals are managed. In this context, metalloproteome plasticity underpinned through the interchangeability of Mn with Mg or Fe in some mononuclear metalloenzymes may play a significant role in some settings. In some cases, this may lead to mixed metal occupancy of enzymes during adaptive processes with potential attendant effects on bacterial fitness, although this will be dependent on the degree to which such enzymes influence metabolic control in a pathway. On the other hand, limited metalloproteome plasticity may be a normal occurrence in cells with no significant selective pressure. The tools to monitor such dynamic changes are becoming available [24,25] and through correlations of metallo-regulatory gene expression, metalloenzyme metal occupancy, ribosome metal composition and cell physiology/metabolic flux a deeper understanding of the pathogen adaptive response during infection should soon be possible.

## Open Access

Open access for this article was enabled by the participation of University of Queensland in an all-inclusive *Read & Publish* agreement with Portland Press and the Biochemical Society under a transformative agreement with CAUL.

## Abbreviations

PPO: PEP–pyruvate–oxaloacetate
Rpe: ribulose-5-phosphate epimerase
